# Patterns of autism spectrum symptomatology in individuals with Down syndrome without comorbid autism spectrum disorder

**DOI:** 10.1186/1866-1955-7-5

**Published:** 2015-01-17

**Authors:** Marie Moore Channell, B Allyson Phillips, Susan J Loveall, Frances A Conners, Paige M Bussanich, Laura Grofer Klinger

**Affiliations:** University of Alabama, Box 870348, Tuscaloosa, AL 35487 USA; MIND Institute, University of California, Davis, 2825 50th St, Sacramento, CA 95817 USA; Ouachita Baptist University, 410 Ouachita St, OBU Box 3734, Arkadelphia, AR 71998 USA; Life Span Institute, University of Kansas, 1000 Sunnyside Ave, Lawrence, KS 66045 USA; Waisman Center, University of Wisconsin-Madison, 1500 Highland Ave, Madison, WI 53705 USA; TEACCH Autism Program; Department of Psychiatry, University of North Carolina at Chapel Hill, CB# 7180, Chapel Hill, NC 27599 USA

**Keywords:** Down syndrome, Autism spectrum disorder, Comorbidity, Social communication, Intellectual disability

## Abstract

**Background:**

Prevalence estimates of autism spectrum disorder (ASD) in Down syndrome (DS) are highly varied. This variation is partly due to the difficulty of screening for and diagnosing comorbid ASD in individuals with a syndrome that carries its own set of social communicative and behavioral difficulties that are not well documented. The aim of this study was to identify the typical range of social communicative impairments observed in children, adolescents, and young adults with DS who do not have comorbid ASD.

**Methods:**

We examined patterns of scores from the five subscales of the Social Responsiveness Scale (SRS) in 46 individuals with DS (ages 10–21 years) without comorbid ASD relative to the published normative sample. We also explored the correlations between SRS symptomatology and age, nonverbal cognition, and receptive language.

**Results:**

SRS scores were elevated (i.e., more ASD symptoms endorsed), with mean scores falling into the clinically significant range. Analysis by subscale revealed a specific pattern, with Autistic Mannerisms and Social Cognition scores significantly more elevated than Social Communication scores, which were significantly more elevated than Social Awareness and Social Motivation scores. Correlations between SRS scores and the other measures varied by subscale.

**Conclusions:**

General elevated ASD symptomatology on the SRS indicates the need for developing population-based norms specific to DS. The pattern of scores across subscales should inform clinicians of the typical range of behaviors observed in DS so that individuals with atypical patterns of behavior can be more easily identified and considered for a full ASD evaluation.

## Background

Down syndrome (DS) is the leading genetic cause of intellectual disability, with an estimated prevalence rate of 8.27 per 1,000 [1]. Historically, researchers have described a certain sociability and friendliness in people with DS, leading to assumptions that they are not impaired in the social domain [2]. More recent research, however, points to a specific set of social impairments and related behavioral challenges in individuals with DS, though these have been understudied [3]. For some individuals with DS, a pattern of atypical social behaviors, along with known cognitive and linguistic impairments [4], could look similar to symptoms of autism spectrum disorder (ASD).

ASD is a neurodevelopmental disorder characterized by impaired social interaction and the presence of repetitive behaviors and/or restricted interests (see [5, 6]). Current estimates of the ASD prevalence rate are approximately 1 in 68, with a male to female ratio of 4–5:1 [7]. With diagnosis limited to behavioral symptoms and the etiology largely unknown, there is extreme heterogeneity within the ASD phenotype [8].

Large-scale population-based studies [9–11] have investigated the prevalence of ASD in individuals with DS, citing rates from 5% to 39% (for reviews, see [12, 13]). Studies comparing individuals with DS and comorbid ASD to those with DS only have found that those with comorbid diagnoses have lower IQ scores [14–16], poorer receptive and expressive language skills [16], and fewer adaptive behavior skills [15–17]. Higher levels of disruptive behavior [18] and stereotypy [9] also have been reported in samples with DS and comorbid ASD relative to DS only. These findings fit within a broader literature suggesting that greater cognitive and/or adaptive functioning impairment increases the likelihood of receiving a comorbid ASD diagnosis for individuals with intellectual disability of various etiologies [19–21]. It is possible, however, that confounding factors such as degree of cognitive impairment, language skills, or adaptive functioning falsely increase the rates of ASD diagnoses in individuals with significant intellectual disability because the currently available measures are not able to accurately capture and separate social, cognitive, linguistic, and behavioral symptoms [12]. Given the potential measurement difficulties, the high variability in rates of diagnosis observed across prevalence studies of comorbid ASD in DS is not surprising.

The heterogeneity within the ASD phenotype requires broad screening measures that capture a wide range of social communication and repetitive behaviors, which may overlap with other disorders such as DS, making it difficult to parse out behavioral symptoms attributable to ASD versus DS [19]. For example, screeners often include characteristics of ASD that are not diagnostic (e.g., certain motor mannerisms, lack of friendships, difficulty switching tasks, or other executive functions), with which many individuals with DS may struggle, even if they do not have ASD, due to their intellectual disability [20, 21]. This is further complicated because researchers are still working to characterize the developmental behavioral phenotype of DS itself [22–24]. Thus, there is an urgent need for ASD screening instruments to be adapted for use in populations with neurodevelopmental genetic disorders associated with intellectual disability, such as DS.

The Social Responsiveness Scale (SRS; [25]) is a commonly used population-based ASD screener that has been normed in the general population. In addition to indicating the overall risk of an ASD diagnosis, the SRS includes subscales that provide a profile of reciprocal social behaviors ranging from normal to severe impairment [25]. These features of the SRS make it a particularly appealing measure to use in populations such as those with DS who may be at risk for comorbid ASD but also likely have a specific profile of strengths and difficulties across domains of social communicative behavior. To our knowledge, however, currently, there are no published data on the SRS in individuals with DS, with or without a comorbid diagnosis of ASD. The present study takes a first step in this direction by providing descriptive data from the SRS in children, adolescents, and young adults with DS who do *not* have comorbid ASD. These data clarify SRS scores that are typical of many individuals with DS and aid clinicians in determining when an individual with DS is at risk for a comorbid ASD.

In the present study, our goal was to examine patterns of symptomatology from the SRS in a sample of children, adolescents, and young adults with DS *without* comorbid ASD. Specifically, we aimed to determine *typical* SRS scores observed in individuals with DS by examining descriptive data from the SRS in our sample relative to the published normative data. Secondary aims were to further characterize the social behaviors associated with DS by examining the pattern of scores across subdomains of the SRS and to explore the relation between ASD symptomatology and age, nonverbal cognition, and language ability in our sample.

## Methods

### Participants

Participants were drawn from a larger study on language development in DS recruited from two university participant registries and via local community agencies, parent support groups, schools, etc. The study advertised recruitment of individuals with DS, ages 10–21 years, who did not have a diagnosis of ASD, who used speech as a primary mode of communication, and who are native English speakers. To be eligible, they were required to have no serious uncorrected sensory or physical impairments that would prevent them from completing the tasks in the larger study (parent/caregiver report). Additionally, to be included in the present study, they had to pass both a hearing screener to rule out uncorrected moderate-to-severe hearing loss and a vision screener to ensure they could adequately see the stimuli presented in the measures. Fifty-four individuals with DS met these criteria and were eligible for the present study.

To ensure that the final sample did not include individuals with comorbid ASD, we used the Social Communication Questionnaire (SCQ; [26]), a caregiver checklist, as an ASD screener. Eight participants scored above our predetermined cutoff of 15 (recommended for individuals with developmental disorders such as DS [26, 27]) and were determined to be at risk for a comorbid ASD diagnosis, and they were referred for a full diagnostic evaluation. Because we intentionally recruited individuals without a diagnosis of ASD, this number should not be used as a prevalence estimate of ASD risk in DS. Although some of the individuals referred for a full evaluation did not receive a diagnosis of ASD, we still excluded them from the present analyses to be conservative^a^.

The final sample size for the present study was 46 (20 males, 26 females; 33 White Non-Hispanic, 7 White Hispanic, 2 African-American, 1 Asian/Pacific Islander, 2 Multi-Racial, 1 not reported). Nine participants (19.6%) had problematic externalizing behaviors (*n* = 4; i.e., ADHD, aggression, property destruction, or tantrums) and/or internalizing behaviors (*n* = 7; i.e., depression, anxiety disorder, or frequent sulking or crying) according to a parent/caregiver-report family background questionnaire. No participants had reported self-injurious behavior or food refusal. Over half of the participants (56.5%, *n* = 26) were on at least one regular medication (parent/caregiver report). The most common type of medication was for hypothyroidism (*n* = 15), followed by ADHD/inattention, allergies/asthma, menstrual regulation, or constipation (*n* = 3 each), followed by heart disease, reflux, or acne (*n* = 2 each). None of the participants had reported seizures. See Table 1 for additional descriptive characteristics of the sample. Ethical approval for human subjects research was granted for this project from the institutional review boards at participating institutions. Parent/guardian consent and child assent were obtained from all participants prior to their inclusion in this project; participants who were of the age of majority signed consent along with their parent/guardian.

**Table 1:** **Sample descriptive characteristics and scores on primary measures**

	Mean (SD)	Range
Chronological age (years)	14.83 (3.27)	10.25–21.92
Nonverbal age equivalent^a^ (years)	5.16 (1.15)	3.42–8.42
SRS total *T*-score	60.57 (11.64)	42–90
SCQ raw score	6.48 (3.63)	1–14
Leiter-R Brief IQ standard score^b^	43.33 (8.41)	36–71
Leiter-R Brief IQ composite growth score	465.07 (10.98)	443–492
PPVT-4 standard score^c^	49.96 (16.77)	20–85
PPVT-4 growth score	140.67 (21.14)	89–179
TROG-2 raw score	27.36 (13.31)	7–72

Leiter-R growth scores are scaled from 372 to 548. PPVT-4 growth scores are scaled from 12 to 271. Sample size was *n* = 46 for Leiter-R, SRS, and SCQ; *n* = 45 for PPVT-4; and *n* = 44 for TROG-2.

^a^Leiter-R Brief IQ age equivalence scores.

^b^11 participants scored at the floor standard score of 36 on the Leiter-R.

^c^3 participants scored at the floor standard score of 20 on the PPVT-4.

### Dependent measure

#### Social Responsiveness Scale (SRS)

The SRS Parent Report Form [25] is a 65-item questionnaire that asks caregivers about their child’s behavior over the past 6 months. Items measure observable aspects of reciprocal social behaviors and cluster into five subscales: Social Awareness, Social Cognition, Social Communication, Social Motivation, and Autistic Mannerisms. Raw scores are converted to *T*-scores (*M* = 50, SD = 10 in the normative sample [28]). Higher scores indicate elevated ASD symptomatology. Scores of *T* ≤ 59 fall within the normal range, *T* = 60–75 in the mild to moderate range, and *T* ≥ 76 in the severe symptom range.

The Social Awareness subscale is defined by Constantino and Gruber [25] as the “ability to pick up on social cues”, with items representing the “sensory aspects of reciprocal social behavior” (e.g., *Seems to react to people as if they were objects*). Social Cognition is the “ability to interpret social cues once they are picked up”, representing the “cognitive-interpretive aspects of reciprocal social behavior” (e.g., *Takes things too literally and doesn’t get the real meaning of a conversation*). Social Communication refers mostly to expressive communication or the “motoric aspects of reciprocal social behavior” (e.g., *Has overly serious facial expressions*). Social Motivation is how the individual is “generally motivated to engage in social-interpersonal behavior” and taps into social anxiety and inhibition (e.g., *Avoids starting social interactions with peers or adults*). Autistic Mannerisms refers to “stereotypical behaviors or highly restricted interests characteristic of autism” (e.g., *Has repetitive, odd behaviors such as hand flapping or rocking)* and also includes items referring to rigidity and inflexibility (e.g., *Has more difficulty than other children with changes in his or her routine*).

Although the SRS was published prior to the changes in ASD diagnostic criteria set forth in the Diagnostic and Statistical Manual (DSM), Fifth Edition [6], the SRS items map on to both the social communication and repetitive behaviors/restricted interests domains of the new two-factor structure [28]. Internal consistency reliability for total SRS scores in the present study’s sample was *α* = .94 for males and .96 for females. Concurrent validity of the SRS was supported in our sample by total scores correlating significantly with SCQ total scores, *r*(44) = .53, *p* < .001.

### Other measures

#### Autism screener

To rule out potential comorbid ASD in our sample, we used the Social Communication Questionnaire-Lifetime (SCQ) [26]. It measures DSM-IV autism symptomatology via parent/caregiver report with 40 items that are formatted as yes/no questions about symptoms present across the child’s lifespan. Scores are calculated out of 39 points, with higher scores indicating greater ASD symptomatology. The SCQ has been studied previously in DS [10, 15, 17, 29] and was found to have good psychometric properties in children and adolescents with DS [15], with a low rate of false negatives [10] but with a potentially high rate of false positives [10, 15]. Although there may be a risk of oversensitivity and modified algorithms may prove useful in the future, there is evidence supporting the use of the SCQ in individuals with DS using a cutoff score of 15 that is recommended by the developers of the SCQ for individuals with developmental disorders [26]. We used this cutoff to exclude participants at or above this score. Reported internal consistency reliability of the SCQ in a typically developing normative sample ranges from *α* = .84 to .93, increasing with age.

#### Nonverbal cognition

The Leiter International Performance Scale-Revised (Leiter-R), Brief IQ [30] is a standardized measure consisting of four subtests of nonverbal cognition that are nonverbal in administration and in participant response method. We modified administration for our sample so that all participants began with Item 1, regardless of chronological age, to ensure that they did not start on an item beyond their ability level.

We used growth scores in the study analyses. Growth scores are scaled corrections of raw scores that take into account item difficulty and are comparable across subtests and ages. Unlike standard scores, growth scores indicate an absolute ability level rather than one relative to age-based norms. Thus, they are psychometrically superior to standard scores for populations with intellectual disability who perform in the lowest potential range of standard scores and often at floor levels [31]. Growth scores also are superior to raw scores because they are scaled. Like raw scores, however, they can be interpreted clinically in terms of age equivalence [30]. For example, a growth score value of 500 on the Leiter-R is comparable to that of the average 10-year-old. Although we used growth scores in the present study’s analyses, for ease of clinical interpretation, we also reported standard scores in the participant descriptives. Reported reliability for the Leiter-R ranges from *α* = .88–.90, and the Brief IQ screener correlates with the Wechsler Intelligence Scale for Children, Third Edition (WISC-III) [32] at *r* = .85. The Leiter-R is normed for ages 2 to 21 years.

#### Receptive language

We created a composite variable for receptive language by converting growth scores from the Peabody Picture Vocabulary Test, Fourth Edition (PPVT-4) [33] and raw scores (growth scores unavailable) from the Test for Reception of Grammar, Second Edition (TROG-2) [34] to *Z*-scores and averaging those values.

The PPVT-4 is a standardized measure of receptive vocabulary normed for ages 2.5–90+ years. Examinees were instructed to point to pictures that best represented words spoken by the examiner. We used growth scores in the analyses but also reported standard scores for clinical interpretation. Reported internal consistency for the PPVT-4 is *α* = .94.

The TROG-2 is a standardized measure of receptive syntax (i.e., the ability to comprehend the grammatical structure and meaning of spoken sentences). Examinees were instructed to point to pictures that go along with sentences spoken by the examiner. The TROG-2 is appropriate for use in ages 4–87 years, though normalized standard scores are not provided for ages 14+; thus, they were not available for the majority of our sample. Because growth score values are not available for the TROG-2, we used raw scores in the analyses. Reported internal consistency reliability for the TROG-2 is .88. There was one statistical outlier on the TROG-2 in our sample; this case was also a bivariate outlier, so we excluded this participant from the correlational analyses.

### Procedure

Each standardized measure was administered to participants in a private room by a trained examiner. As part of the larger study, the Leiter-R and TROG-2 were administered during the same session, and the PPVT-4 was administered in a separate session. Parents/caregivers completed the SRS and SCQ prior to the child sessions.

## Results

See Table 1 for descriptive statistics. There were no major violations of normality, and all assumptions were met for the statistical analyses.

### SRS scores in DS: comparisons to the normative sample

Group-wise, participants showed elevated overall SRS scores, indicated by the mean total *T*-score of 60.57 (SD = 11.64), which is just above the normal range and into the clinically significant “mild-to-moderate” category of ASD symptomatology. Further, a one-sample *t*-test comparing participants’ *T*-scores to the mean value (*T* = 50) from the normative sample was significant, *t*(45) = 6.16, *p* = < .001.

**Figure 1:**
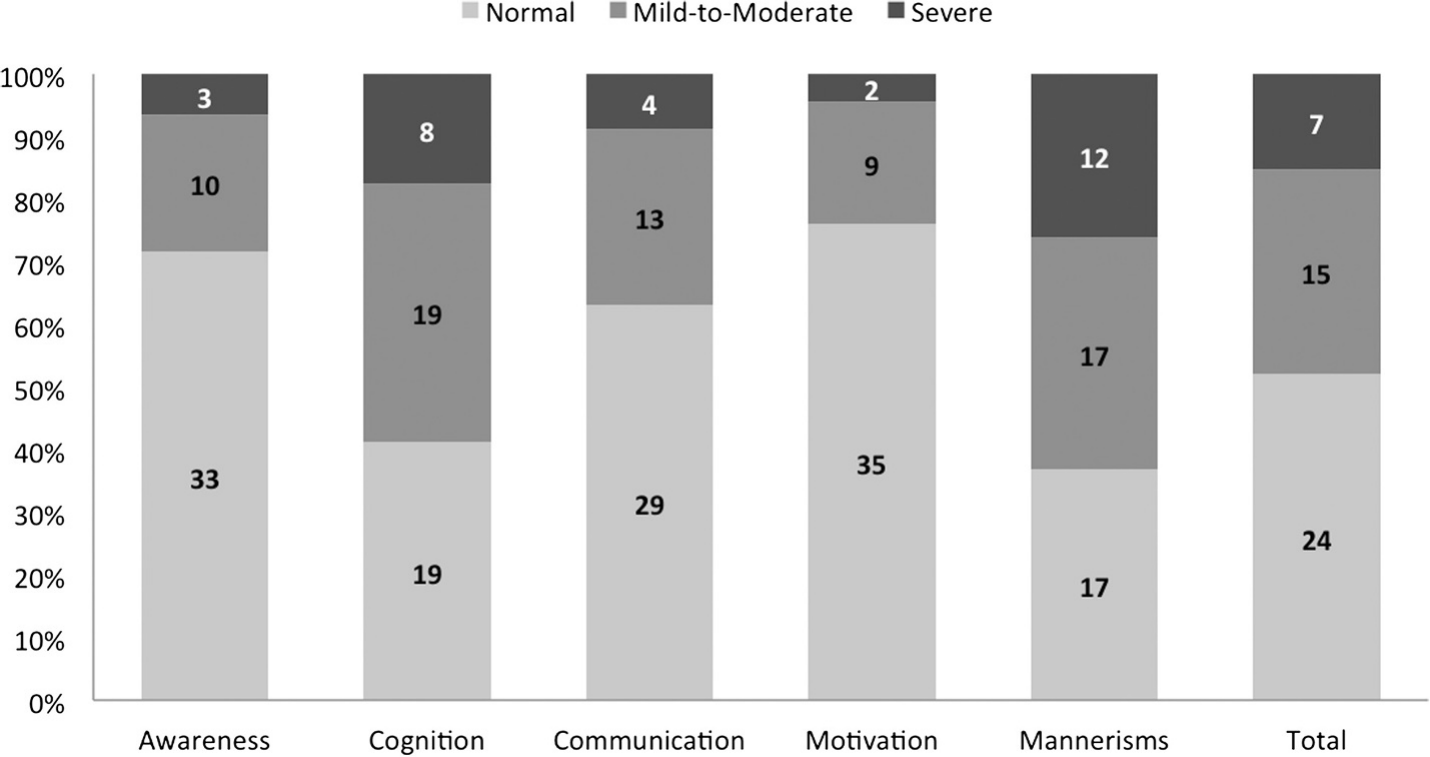
**Percentage of participants falling into the “normal”, “mild-to-moderate”, and “severe” categories on the Social Responsiveness Scale (number of participants also provided).**

An examination of SRS *T*-scores by subscale revealed that the mean *T*-scores for Autistic Mannerisms and Social Cognition were beyond the normal range cutoff of *T* = 60. The mean scores for the other subscales were just below this cutoff, with the range of scores spanning above and below 60 for every subscale. See Figure 1 for the distribution of *T*-scores into each clinical category by subscale. One-sample *t*-tests comparing participants’ subscale *T*-scores to the mean value (*T* = 50) from the normative sample were all statistically significant such that, for each subscale, the mean score of study participants was significantly higher than that of the normative sample (Table 2).

**Table 2:** **One-sample** ***t*** **-tests comparing SRS subscale** ***T*** **-scores to the mean value (** ***T*** **= 50) from the normative sample**

	Study sample mean (SD)	***t***-statistic	***p***value
Social Awareness	54.04 (11.54)	2.38	.022
Social Cognition	63.22 (12.51)	7.16	<.001
Social Communication	58.33 (11.08)	5.10	<.001
Social Motivation	53.80 (11.42)	2.26	.029
Autistic Mannerisms	65.07 (13.51)	7.56	<.001

The normative sample standard deviation (SD) = 10 for each subscale of the SRS.

### SRS scores in DS: within-group comparisons

We used a 2 (sex) × 5 (subscale) mixed ANOVA to examine the pattern of scores across SRS subscales within the present study’s sample, with sex included as a between-subjects factor. The ANOVA revealed no significant main effect of sex, *F*(1,44) = 0.42, *p* = .52, *η*_p_^2^ = 01. There was a significant main effect of subscale, *F*(4,41) = 15.01, *p* < .001, *η*_p_^2^ = .59. Fisher’s LSD *post hoc* analyses showed that Autistic Mannerisms and Social Cognition *T*-scores were significantly more elevated than Social Communication scores, which were significantly more elevated than Social Awareness and Social Motivation scores. The interaction term was not significant, *F*(4,41) = 0.68, *p* = .61, *η*_p_^2^ = .06. Every subscale was significantly correlated with all the other subscales at *p* < .01, with Pearson’s *r* values ranging from .44 to .74 (Table 3).

**Table 3:** **Correlations among SRS subscale** ***T*** **-scores**

	Awareness	Cognition	Communication	Motivation	Mannerisms
Awareness	-				
Cognition	.62*	-			
Communication	.74*	.74*	-		
Motivation	.44*	.52*	.63*	-	
Mannerisms	.56*	.65*	.66*	.46*	-

*All correlations were significant at *p* < .01.

### SRS scores in DS: relations with other sample characteristics

We explored the relations between SRS ASD symptomatology and age, nonverbal cognition, and receptive language in our sample. Results indicated that age was not significantly correlated with SRS total or subscale *T*-scores. Nonverbal cognition, however, was negatively correlated with SRS total scores and Autistic Mannerisms and Social Cognition subscales^b^. Receptive language was negatively correlated with SRS total and all subscale scores (Table 4).

**Table 4:** **Correlations between SRS and age, nonverbal cognition, and receptive language**

	SRS ***T***-scores					
	Total	Awareness	Cognition	Communication	Motivation	Mannerisms
Age^a^	-.003	-.10	.05	-.04	.03	.04
Nonverbal cognition^b^	-.30*	-.27	-.33*	-.21	-.15	-.30*
Receptive language^c^	-.46*	-.43*	-.38*	-.34*	-.30*	-.48*

*p < .05.

^a^Age in years (*n* = 45).

^b^Leiter-R growth scores (*n* = 45).

^c^PPVT-4 and TROG-2 *Z*-score composite (*n* = 44).

## Discussion

We identified patterns of ASD symptomatology, measured by the SRS, in individuals with DS who do not have comorbid ASD. Secondary aims were to characterize the profile of reciprocal social behaviors captured by the subscales of the SRS in individuals with DS and to explore the relationship between ASD symptomatology and other participant characteristics within our sample.

In the primary aim, after excluding comorbid ASD, our conservative sample of individuals with DS showed significant elevations on all subscales of the SRS relative to the population-based normative sample. Their mean total *T*-score surpassed the cutoff for clinical significance, falling into the mild-to-moderate symptom severity category. Despite their lack of comorbid ASD, ASD-like symptoms were present at higher rates group-wise in our sample than in the typical population. Thus, there may be a tendency for the SRS to produce false positives when used as an ASD screening tool in individuals with DS. Because the SRS is commonly used in clinical settings, our findings emphasize the need to obtain normative data on the SRS for DS and other atypical populations. Such an endeavor will require a large-scale project beyond the scope of the present study.

The next aim was to characterize the profile of reciprocal social behaviors associated with DS. We accomplished this by examining the pattern of scores across the subscales of the SRS within our sample. The participants showed an uneven profile such that their scores were most elevated (i.e., more ASD-like symptoms) for Autistic Mannerisms, followed by Social Cognition and Social Communication, and their scores were lowest (i.e., fewer ASD-like symptoms) for Social Awareness and Social Motivation. If confirmed by future studies, this pattern suggests that an examination of SRS scores by subscale could be useful in deciding whether to refer an individual with DS for a comprehensive ASD diagnostic evaluation, as the appropriate cutoff for considering an individual at risk may vary by subscale.

The finding that participants exhibited many behaviors associated with autistic mannerisms (stereotypical behaviors, highly restricted interests, rigidity, and inflexibility) is noteworthy and suggests that some of these behaviors may be more common to the general DS phenotype and are not necessarily indicative of comorbid ASD in this population, at least for the age range of the present study’s sample. Indeed, others also have noted the presence of repetitive behaviors in other developmental disorders, including DS (see [35] for a review).

The pattern of symptomatology across the other SRS subscales suggests that despite a general orientation toward social-interpersonal behavior (Social Motivation) and an awareness during social interactions that allows individuals with DS to use appropriate social cues (Social Awareness), they exhibit relatively greater difficulty interpreting social cues exhibited by others (Social Cognition) and in nonverbal expressive communication (Social Communication). Social Awareness items on the SRS measure a general awareness of common social rules (e.g., modulating volume of voice, not interrupting others, making appropriate facial expressions). Social Cognition items, however, require accurate interpretation of a social partner’s verbal and nonverbal cues and sometimes require understanding another’s intentions (e.g., understanding others’ humor or sarcasm, knowing when someone is being unfair). The pattern observed in our sample is generally consistent with literature from behavioral studies (see [3, 23, 36, 37]) and is relevant to the social behavioral phenotype of DS. It is important to remember that, despite the patterns of relatively more or less elevated domains of autism symptomatology within-syndrome, at the group level, participants showed significantly elevated scores across all domains relative to the typical population.

Our final aim was to explore the relation between ASD symptomatology and other participant characteristics. Lower receptive language ability was related to elevated symptomatology for all SRS subscales. Lower nonverbal cognitive ability also was related to elevated symptomatology, but this relation was only significant for the Social Cognition and Autistic Mannerisms, the two subscales for which participants displayed the most elevated scores. These findings extend prior research on the relation between ASD symptoms, cognition, and language in individuals with DS and comorbid ASD [14–16] by demonstrating a similar link in a sample with DS only. They also raise the concern that the SRS may especially over-identify ASD in those who have DS and significant delays. The observed correlations, however, were only modest in strength, and the sample size was limited. Thus, more research is needed to replicate these findings in larger samples and to determine whether they are unique to DS or characteristic of other genetic syndromes associated with intellectual disability by directly comparing such samples.

If upheld by future research, the association between ASD symptoms and language ability is particularly important given the core language difficulties exhibited by many people with DS [4]. It means that the individuals with DS who struggle most with understanding verbal communication by others are more likely to exhibit atypical social behaviors when interacting with others, even in the absence of comorbid ASD. Regardless of the explanation, it demonstrates a need for promoting social development, in particular social information processing and nonverbal communication, in DS along with speech and language services. Improving social communication skills would give individuals with DS a better chance of behaving and responding to others in socially appropriate ways, thus increasing the likelihood of establishing and maintaining positive interactions with peers [38, 39].

### Limitations and future directions

Although the present study takes a significant step toward addressing the issues related to screening for comorbid ASD in individuals with DS, it has several limitations. First, our sample was not fully representative of the broader population with DS. Instead of a population-based sampling approach, this sample was drawn from a behavioral research study for which participants had to meet several study-relevant criteria to participate. However, based on their Leiter-R nonverbal IQ and PPVT-4 scores, our sample appears comparable to samples reported in most other behavioral research studies of youth with DS [4]. Additionally, we excluded individuals with DS who have comorbid ASD. Future studies should recruit individuals with DS more broadly to obtain a more representative sample and directly compare those with and without comorbid ASD. Future studies also should consider controlling for comorbid medical conditions (e.g., medication use, chronic sleep disturbance) and other variables (e.g., social anxiety or avoidance) that may impact behavior and ASD symptomatology.

Second, our sample size was small, especially given the heterogeneity of DS. The analyses failing to detect a significant effect (i.e., main effect of sex and the interaction effect involving sex in the ANOVA), however, revealed quite small effect sizes, strengthening the conclusion that there was no significant between-subjects difference. Further, in the exploratory correlational analyses, although we only had enough statistical power to detect moderate to large correlations, the non-significant correlations with age were very small (.003–.04). Despite our confidence in the statistical interpretations, the small sample size across such a wide age range of participants limits our ability to interpret the relation between ASD symptomatology and age. Third, our study was limited to caregiver report of ASD symptomatology. Future studies should include direct observation and behavioral assessment to provide a more complete description.

Finally, because our study only included individuals with DS ages 10–21 years, the clinical implications for individuals outside of this age range are limited. In particular, a similar study is needed in younger children with DS to strengthen the efforts to screen for and identify ASD in early childhood. Longitudinal studies will prove particularly informative as it is likely that the profile of reciprocal social behaviors associated with DS and its relation to other characteristics (e.g., nonverbal cognition and language ability) evolves with age.

## Conclusions

The present study is the first to report data from the SRS, a commonly used ASD screening tool, in a sample with DS without comorbid ASD. In general, scores were elevated relative to the available normative data on typically developing children and adolescents, suggesting the need for normative data on the SRS for syndrome-specific samples such as those with DS. This would improve specificity of the instrument as a screening tool for ASD and minimize the number of individuals with DS unnecessarily referred for full ASD diagnostic evaluations. This is particularly important as the diagnosis of ASD in DS is becoming a greater focus of clinicians and researchers alike. Also needed are more empirical data on ASD diagnostic tools in samples with DS. It is likely that some of the currently used diagnostic measures also will require modification for ease of use and interpretation in the DS population. Finally, more research is needed to detail the social behavioral phenotype of DS from a developmental perspective. Until the emerging DS phenotype is more fully understood, distinguishing between typical and atypical social behaviors within this disorder will remain a challenge.

## Endnotes

^a^A more conservative SCQ cutoff of 13 resulted in the same pattern of findings for all study analyses (*n* = 43). ^b^Despite the observed floor effects on Leiter-R standard scores in our sample, the pattern of results did not change when using standard scores rather than growth scores in the correlational analyses.
